# Understanding networking behaviors among Indian startups: A qualitative study

**DOI:** 10.12688/f1000research.159752.1

**Published:** 2025-03-10

**Authors:** Sheetal Singh, Savitha Basri

**Affiliations:** 1 commerce, Manipal Academy of Higher Education, Manipal, Karnataka, 576104, India; 2 Department of Commerce, Manipal Academy of Higher Education, Manipal, Karnataka, 576104, India

**Keywords:** Entrepreneurship, Formal Networking, Informal Networking, Networking Behavior, Startups, Experience, India

## Abstract

**Background:**

Networking plays a pivotal role in achieving business success and mitigating startup failures. The purpose of this qualitative study is to explore the networking experience of startup entrepreneurs for the growth of their businesses.

**Methods:**

A qualitative semi-structured interview was conducted mainly in Bangalore and the Delhi regions of India. Using a cluster sampling method, interviews were held with 36 startup entrepreneurs. These startup owners were mainly from Bangalore and Delhi. The age group, gender, demographic profile, and popular startup sector of these startup owners have also been analyzed in this study. The data were analyzed using Atlas. Ti software.

**Results:**

The qualitative findings revealed four overall themes related to networking, namely, the “role of networking for the success of the startups”, “supportive environment for networking”, “crucial stages of networking” and “role of past working experience of entrepreneurs”. It was found that most entrepreneurs leverage past work experience to interact effectively in business, utilizing skills such as communication, leadership, and market intelligence.

**Conclusion:**

Entrepreneurs mainly prefer networking with colleagues and their ex-bosses in building and creating formal networking. During crises, network members provide expert advice, moral support, and connections to specialists, which helps them solve problems and access new opportunities. Networking is crucial mainly in the early stage of a startup, where connections and relationship structures are needed for navigating upcoming challenges.

## 1. Introduction

Entrepreneurs are generally socially oriented individuals who are actively involved in community affairs. They also possess a strong sense of global trends and market opportunities. Additionally, these successful entrepreneurs prioritize building social trust among their colleagues and business partners, which leads to broad support and mutual benefits. In today's highly competitive business environment, networking stands out as an essential tool for achieving success. Networking is crucial for startups to grow and thrive. It is a process of building relationships that work together to enhance. Networking is a way to reduce the risk of a new firm by improving its credibility and alleviating some of the problems experienced by startups, such as lack of funds, workplace issues, technology support, mentoring, expertise, marketing strategy, sales difficulties, and capital allocation issues ( Albourini *et al*., 2020; Ferreira *et al*., 2022).

Networking can involve one-time or continuing interactions and can be planned or unexpected. It involves the formation and maintenance of a variety of inter-organizational social ties depending on the needs of startups. Partnerships and familial ties are examples of these networks that have an impact on decision-making and business efficiency. These networks help the entrepreneur's business in a variety of ways, including business growth, partner discovery, taking advantage of the right opportunity at the right time, and increasing visibility. Making relationships with the right people at the right time is important in networking. The survival and success of a startup depend upon the networking habits of the startup founders. Establishing and nurturing relationships with professionals across various industries enables business owners and professionals to access valuable resources, information, and opportunities. Networking serves as a means to expand knowledge, keeping individuals abreast of industry trends. Moreover, it can lead to valuable business partnerships, collaboration, and referrals, ultimately enhancing business outcomes. By tapping into a broad network of potential clients, investors, and partners, networking can significantly contribute to increased sales and revenue ( Ibrahim & Roslin, 2018). Networking also provides a platform for skill development and learning from industry experts and peers. Overall, it plays a pivotal role in broadening reach, gaining valuable insights, building strategic alliances, and driving growth and success.

Networking behavior in business refers to intentional actions aimed at establishing and nurturing connections to achieve specific business outcomes. Actively engaging in networking behaviors, such as cultivating internal and external contacts, participating in professional and community activities, and raising one's profile within the company, allows organizations to expand their network and access diverse opportunities. This strategic approach enables businesses to leverage relationships and social connections for a competitive advantage, leading to various positive business outcomes ( Albourini *et al*., 2020).

Previous research has primarily focused on the role of networking in the early stages of business development, such as startup formation and initial growth. There is limited research on the challenges faced by entrepreneurs and startups in sustaining and expanding their networks as they progress beyond the startup phase ( Albourini et al., 2020; Ferreira et al., 2022). A review of existing studies reveals that most are predominantly quantitative, relying heavily on questionnaire-based methodologies. However, there is a noticeable gap in understanding entrepreneurs' perceptions of networking behavior and its impact on their startups. This study seeks to bridge this gap by enriching the existing knowledge body and offering a fresh perspective on entrepreneurial networking practices. This research gap highlights the need for further investigation into the various aspects of networking in the context of startups, including understanding the role and impact of effective networking strategies, identifying supportive network members, exploring the timing and sequence of networking activities, and examining the influence of past working experience in the context of startups. The objective of this study is to understand the entrepreneur's experience and examine the networking behavior of startups. An exploratory method was used to analyze the most important themes and concepts that drive networking behavior in Indian startups to do this. The paper is segmented into five main areas including literature review, methodology, findings, management implications, limitations, and future research directions.

## 2. Literature Review

Social networks affect entrepreneurial behavior, signifying that entrepreneurs with robust social connections are inclined to actively participate in entrepreneurial activities. Moreover, networking also provides startups with a platform to showcase their products or services, increasing visibility and attracting potential customers or clients ( Anderson *et al*., 2010; Ferreira *et al*., 2022). By networking startups can establish a strong support system and increase their chances of success in a competitive business environment ( Witt, 2004; Ferreira *et al*., 2022). The development of relationships both inside and outside the company as well as participation in professional events are examples of networking behaviors that have a favorable impact on the success of entrepreneurial ventures. The networking behavior that has the biggest influence on the success of entrepreneurial startups is cultivating external contacts ( Albourini et al., 2020). In entrepreneurial incubation hubs, networking promotes the growth of start-ups by exchanging ideas and experiences. Networking is essential for promoting the entire incubation process and helping start-ups grow through the exchange of ideas and experiences. Startups must first establish a network to work together successfully ( Ferreira et al., 2022). Through the infusion of ideas and experiences, networking plays a critical role in facilitating the overall incubation process and assisting start-ups in their development. Start-ups can only successfully collaborate if they begin to network ( Karambakuwa and Bayat, M. S. 2023).

Strong and weak ties on social media offer various forms of support, which is crucial for the growth of startup businesses. The most helpful contacts for startups were friends, business angels, and business partners, but they also leverage weak ties to get different kinds of help. Although business incubators were utilized by a large number of entrepreneurs, their assistance in creating external networks and connections was not well recognized ( Durda, L., and Ključnikov, A. 2019). Formal and informal networking is crucial to the success of a startup ( Sharafizad, 2014). Formal networking refers to intentional and structured efforts to establish connections with other professionals and organizations ( Witt, 2004). On the other hand, informal networking refers to the natural and spontaneous relationships that develop through day-to-day interactions and personal connections. These informal networks often include friends, family, colleagues, and mentors who can provide support, advice, and potential business opportunities ( Abou-Moghli & Al-Kasasbeh, 2012; Albourini *et al*., 2020; Gloor *et al*., 2018; Ferreira *et al*., 2022). The combination of both formal and informal networking can provide startups with a wide range of benefits. Formal networking allows startups to expand their professional network, connect with industry leaders and experts, and gain access to valuable resources such as funding opportunities, partnerships, and market insights. On the other hand, informal networking helps startups build relationships based on trust and personal connections. These relationships can provide emotional support, mentorship, and access to informal knowledge and advice. Additionally, formal networking can provide startups with credibility and visibility within their industry, enhancing their reputation and attracting potential customers or clients ( Klerk & Saayman, 2012; Sharafizad, 2014; Stuart & Sorenson, 2007). On the other hand, informal networking offers startups a support system of trusted individuals who can provide guidance, feedback, and referrals ( Sharafizad, 2014; Witt, 2004).

Networks play a critical role in the performance of small businesses during both their startup and expansion phases. A study discovered a favorable correlation between small business performance throughout both the startup and expansion phases and the utilization of entrepreneurial networks. As a company advances from the start-up to the growth stage of the business life cycle, small business performance improves Tendai, C. 2013. In the early stage, networks are crucial for information and resource access, and performance is positively impacted by these networks ( Tendai, 2013). The development of critical capabilities through networking is particularly important in the founding, start-up, and production phases ( Laurell *et al*., 2017). As startups expand, strategic alliances and specific ties become more beneficial ( Chu and Yoon, 2021). However, the size and occupational background of the network can also influence the success of the startup ( Greve, 1995). The importance of establishing strong relationships at the beginning of network formation is emphasized ( Jørgensen and Ulhøi, 2010). Depending on their business phase and startup incentives, women entrepreneurs have different networking behaviors and structures within their networks. In the pre-startup stage, there were no variations in the networking practices and frameworks of the various categories of female entrepreneurs; nevertheless, during the startup and established stages, disparities surfaced ( Sharafizad and Coetzer, 2017).

Prior experience of entrepreneurs is positively correlated with business performance in a minor but significant way, with the experience and cultural context acting as moderators ( Jiao et al., 2023). Previous work experience and the success of entrepreneurial enterprises were found to be positively correlated by a study ( Siddique et al., 2022). An entrepreneur's decision to pursue a business takeover or launch a new firm is influenced by their prior job experience. Starting a new business as a means of pursuing entrepreneurship is more likely when one has both management experience and experience in the same industry ( Xi et al., 2018). The previous experience of entrepreneurs allows them to identify opportunities, anticipate challenges, and make informed decisions ( Dorcas *et al*., 2021). Work experience equips entrepreneurs to navigate the challenges of running a business and increase their chances of success ( Lee & Tsang, 2001). Prior work experience plays a significant role in an entrepreneur's skills and knowledge, influencing his or her ability to recognize and act on opportunities, manage new ventures, and address the changing role of a business founder ( Martin and Smith, 2010). This experience can also decrease uncertainty and motivate entrepreneurs ( Othman *et al*., 2016).

## 3. Methods

### 3.1 Study design and setting

This study adopted qualitative research methods by conducting semi-structured interviews with 36 startup entrepreneurs based in the Bangalore and Delhi regions in India. The startup owners constitute a higher percentage of males as compared to female startup owners in this study. 

This study developed and implemented an intervention grounded in a culturally sensitive framework, drawing on the recommendations of Gibbons et al. (2019) and McWhirter et al. (2013, 2019). The study's objectives shaped the choice of a qualitative methodology, aligning with a constructivist paradigm ( Allen, 1994). This approach emphasizes interpreting and contextualizing the meanings derived from entrepreneurs' experiences, enabling an in-depth investigation of how networking behavior among Indian startups contributes to their business success. Recognizing the potential impact of networking behavior on entrepreneurial success, a comprehensive examination of this phenomenon became imperative. Semi-structured interviews were chosen as the preferred data collection method due to their flexibility, which enables deeper exploration of topics that may surface unexpectedly and require further investigation during the interview. Given the limited research in this area, these interviews allowed for a more comprehensive understanding of entrepreneurs' experiences. To delve deeper into this aspect, questions about the networking process were methodically integrated into the topic guide during the iterative data collection phase of our mixed methods study. The formulation of these inquiries was informed by insights gleaned from initial interviews, thus ensuring a nuanced exploration of networking dynamics within the entrepreneurial context. The institutional Ethics Committee granted ethical approval for the study, and written informed consent was obtained from participants before the interviews began. Some interviews were recorded in audio files, and some responses were noted for taking interviews. All the interviews were conducted from May 2023–July 2023. Given the aim of this study—to explore entrepreneurs’ thoughts and preferences regarding networking behavior in Indian startups—conducting in-depth interviews was deemed the most appropriate approach to address the research questions.

### 3.2 Interview guide

These interview questions were asked to the participants about their experiences in the networking process ( Table 1). The focus of the interviews centered on entrepreneurs' networking behavior and subsequent business outcomes. The average length of the interviews ranged from 20 to 30 minutes.

**Table 1.** An interview guide for exploring participants’ experiences in networking behavior.

Questions	Prompts
Is there any role of networking in the success of your start-up?	What Are They?
Does networking help you to get support from other start-up owners?	Positive/Negative Aspects?
Is your past work experience helping you interact with more people?	Yes- How/No
How do you collect information related to business while interacting with others?	Laptop, Book, Mind, No Need, Google Key
Who do you look for in your guidance and mentorship?	Family, Friends, Or Outsiders
With whom you would like to maintain the networking more (i.e., a known group?	Family, Friends, Or Outsiders
Which network is important in getting more business opportunities?	Team/Informal and Others
Do network members help you in facing business crises or problems?	If Yes, How Do They Support and Help You?
At what stage of business do you think networking is important for you?	Early, Expansion, Growth, and Decline
What motivated you to start networking and expand your network?	Finance, Competition, Growth

### 3.3 Tools for analyzing data

Thematic analysis was selected as the method of analyzing the interview transcripts, given that it is a flexible approach that can accommodate diverse ontological and epistemological perspectives ( Braun & Clarke 2006). Thematic analysis is a systematic framework for coding qualitative data to identify meaningful patterns across the data ( Braun & Clarke, 2014). Thematic analysis is a particularly popular approach to qualitative research with some areas of application that crossover towards other kinds of applied policy and practice-focused discourse, not dominant in the academics ( Braun & Clarke, 2014). The thematic analysis consists of several distinct steps, such as familiarization with the data, generation of initial codes followed by searching for them, and reviewing to then define themes ( Braun & Clarke, 2006). Field notes and reflexive memos were written immediately after each interview, which was the unit of data collection. The data were coded initially and as each stage of the coding progressed from initial to developed themes. We used Atlas. Ti software for the data analysis. We coded the data in ATLAS.ti 23.2.1 for Mac and produced the following four themes from our analysis.

(ATLAS.ti Scientific Software Development GmbH. (2023). ATLAS.ti Mac (version 23.2.1) [Qualitative data analysis software]. https://atlasti.com). Data analysis can also be done by various alternative software, such as QDA Miner Lite ( https://provalisresearch.com/products/qualitative-data-analysis-software/freeware/), and Gephi ( https://gephi.org/). These software also includes features like coding, annotation, and visual analysis).

## 4. Results

Startup owners who are between 30-50 years old represent the largest demographic profile, followed closely by those over 50 years. Conversely, the youngest age group, under 20 years, constitutes a lower percentage in the study. The startup age is between one to five years of operating a startup, some of the startups are based on Fintech, Edtech, and Healthtech. The Fintech and Health sectors seem to be a popular choice for startup sectors, followed by the education sector. Networking across various sectors and business models is vital for survival, growth, and legacy planning. Four main themes during the interviews and after the coding analysis of the interviews.

Theme 1- Role of networking in the success of startups

Theme 2- Supportive environment and people for networking

Theme 3- Crucial stages of networking

Theme 4- Role of the past work experience of an entrepreneur

Each theme is analyzed explicitly with the support of past literature, references, and a network diagram to gain deeper insights into this study.

### 4.1 Theme 1: Role of networking in the success of startups

**Figure 1.**
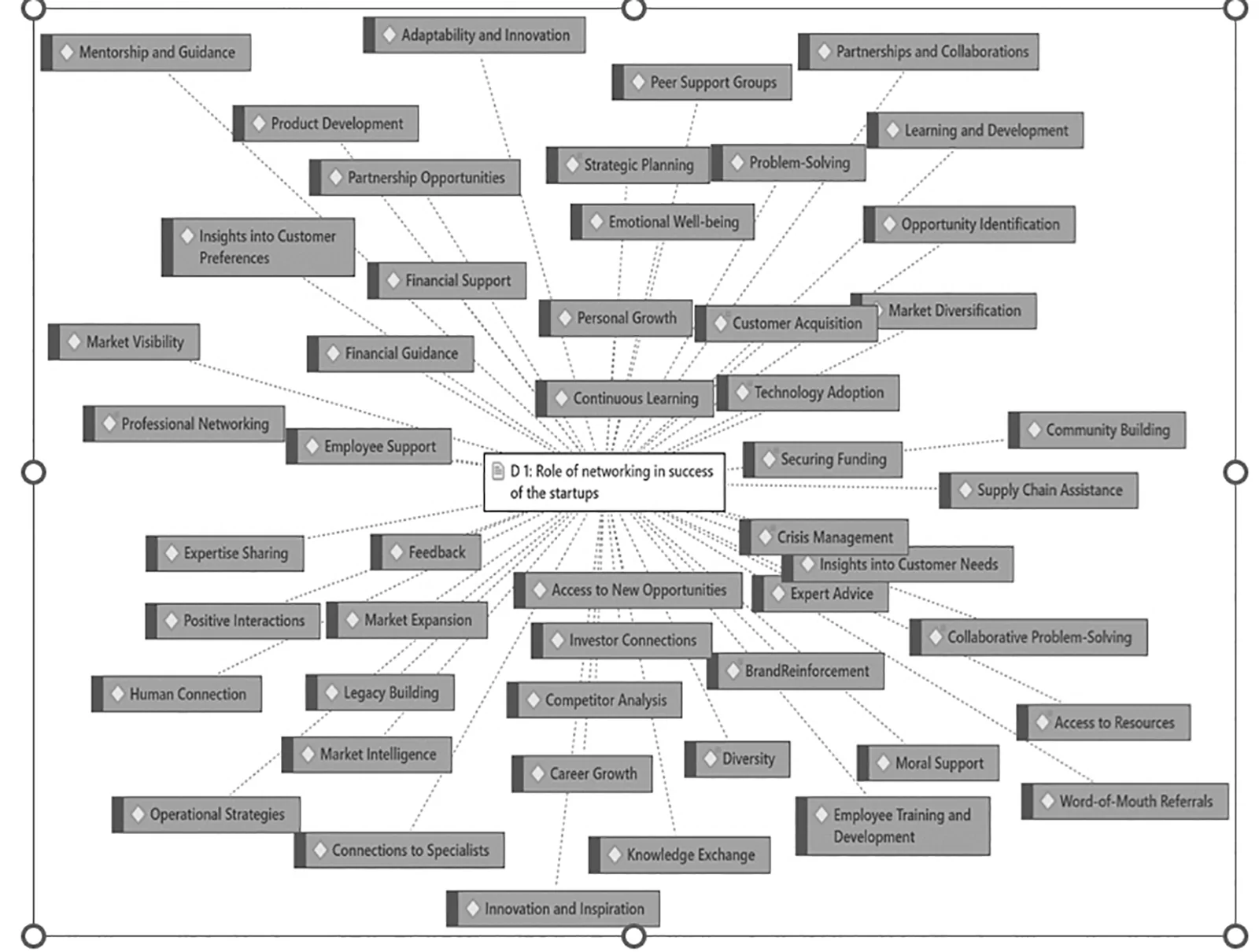
Role of networking in the success of startups. Source: ATLAS. Ti thematic map.

Based on the insights gathered from the interviews, some of the references are mentioned below with a thematic map, as provided in Figure 1. Networking serves as a multifaceted tool that contributes significantly to various aspects of a startup's journey. It facilitates product development by providing avenues for collaboration, feedback, and idea exchange with industry experts, potential customers, and fellow entrepreneurs. This collaborative environment fosters innovation and helps refine the startup's offerings to better meet market needs.

Networking helps startups obtain financial support. By connecting with investors, venture capitalists, and other funding sources, entrepreneurs can secure the necessary capital to fuel their growth and expansion plans. It also helps in problem-solving and allows entrepreneurs to tap into the collective expertise and experiences of their network to overcome challenges and navigate obstacles more effectively. Additionally, networking provides new opportunities for startups, including potential collaborations, joint ventures, and market expansion prospects. By expanding their professional network, entrepreneurs increase their chances of discovering and capitalizing on these opportunities.

Reference 1- ‘… *We collaborate with like-minded professionals with whom we feel comfortable and aligned. Our ability to assemble teams on a project-by-project basis not only meets the demands of our current endeavors but also facilitates the expansion of our business capacity* …’.

Reference 2- ‘…. *Networking transcends merely exchanging business cards; it is about building meaningful connections. Given the impracticality of following up with everyone met at these events, selectivity becomes crucial. Instead of spreading oneself thin, it is more effective to prioritize contact with individuals deemed valuable for maintaining ongoing relationships ….’.*

Reference 3- ‘… *Well, I wanted to make my career grow until I retire that’s what everybody wants even if they’re working for someone else. I also get a lot of satisfaction out of working for myself ….’.*

### 4.2 Theme 2: Supportive environment and people for networking

**Figure 2.**
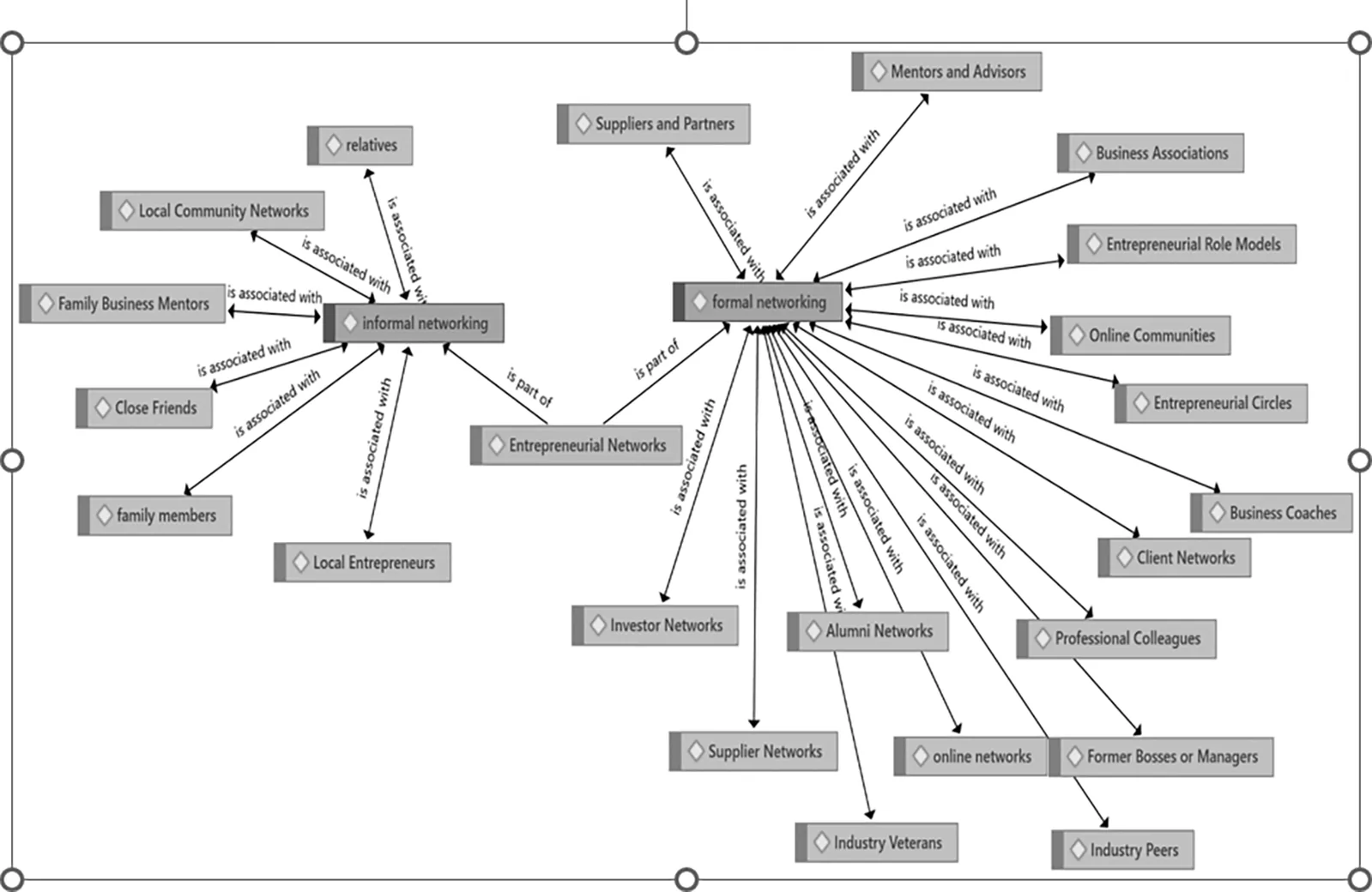
Insights into supporting members for networking. Source: ATLAS. ti thematic map.

Drawing from the insights obtained from the interviews, a selection of references is included below, accompanied by a thematic map, as shown in Figure 2. This shows that collaborating with peers can lead to innovative solutions and enhanced customer experiences while also attracting top talent and increasing brand visibility. Industry events and conferences provide face-to-face networking opportunities and access to industry experts and potential collaborators. Alumni networks from educational institutions can serve as a valuable resource for making connections with fellow entrepreneurs who share similar backgrounds and experiences. Additionally, business mentor networks offer access to seasoned professionals who can provide guidance and support based on their entrepreneurial journeys.

Reference 1- ‘. *We maintain enduring business relationships with partners we have engaged with for over two decades—individuals we trust implicitly. Their track record of consistent delivery is a testament to their reliability. Despite potential cost differentials, our confidence lies in their steadfast commitment to honoring their words. This trust ensures peace of mind, knowing that they will follow through on their promises* …’.

Reference 2- ‘… *I consulted with a select group of individuals, including friends, family members, and my then-husband, before finalizing my decision …’.*

Reference 3- ‘…. *I maintain a close circle of friends with whom I frequently engage in discussions about my plans and decisions. Their guidance and support have been invaluable to me, and I continue to rely on them as a trusted informal source of advice ….’.*

Reference 4- *‘… I reached out to a friend who operates a similar business, and she graciously provided me with valuable advice and insights. Since our venture did not require a substantial loan from a bank, we utilized our existing capital to establish it and proceeded from there. We spread the word about our business among friends, fellow school moms, and through word-of-mouth marketing channels ….’.*

Reference 4- *“My accountant attends several seminars and keeps me informed about any changes in regulations or laws that may impact my business. With this arrangement, I rely on their expertise to stay up-to-date, allowing me to focus on other aspects of running the business ….’.*

Reference 5- ‘…. *Most of my business derives from informal networking and word-of-mouth referrals. As a small-scale enterprise, I find little necessity to attend to formal business functions. Instead, my primary source of clientele stems from connections within the school mom community ….’.*

### 4.3 Theme 3: Crucial stages of networking

**Figure 3.**
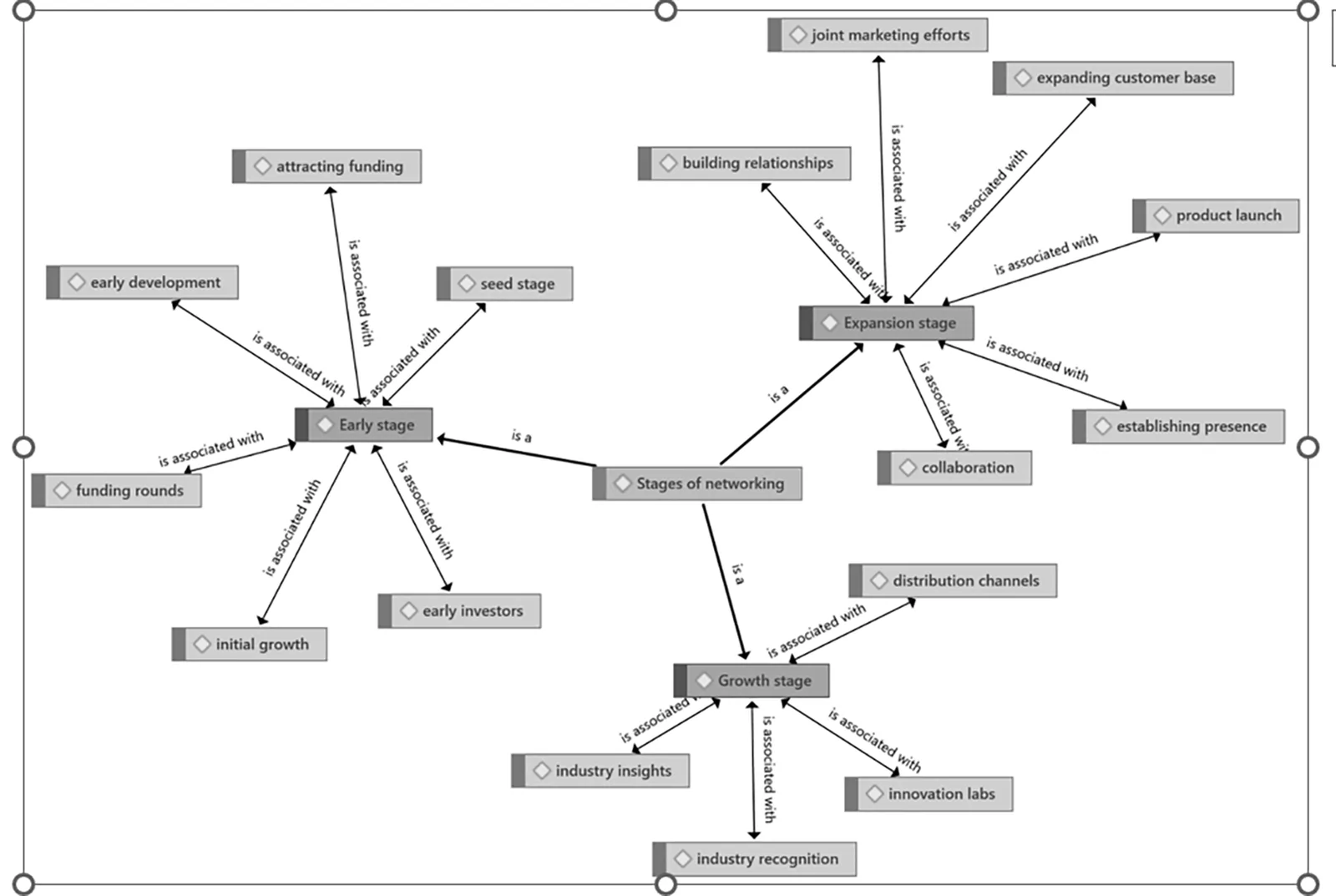
Need for networking at different stages of startup. Source: ATLAS. Ti thematic map.

The participants' view on the crucial stages of networking in the success of the startups has pointed out certain things mentioned below in the references ( Figure 3). At the seed stage, networking helps in establishing initial connections with potential partners, mentors, and investors. These connections are instrumental in gaining market feedback, refining business ideas, and attracting the necessary resources to kickstart operations. As the business progresses, networking becomes essential for securing partnerships, expanding the customer base, and accessing new markets. It also aids in scaling operations efficiently, attracting funding, and gaining industry recognition, positioning the business as a market leader. Ultimately, networking is an ongoing effort that provides valuable opportunities and support throughout the business lifecycle. By actively engaging with various stakeholders, entrepreneurs can leverage their networks to drive growth, innovation, and resilience in the face of challenges.

Reference 1- *‘…. In the initial stages of starting up, I dedicated significant time to conducting thorough research. I reached out to diverse organizations and engaged in discussions with numerous professional contacts both within and beyond my community ….’.*

Reference 2- *‘….During our initial stages, we dedicated significant effort to attending numerous network functions and building relationships within the community to establish our presence. However, our reliance on such activities has diminished as our business has become more established. We now prioritize networking efforts primarily when exceptional opportunities arise, ones that we believe could significantly benefit our business or align with our goals ….’.*

Reference 3- ‘…. *Mainly networking is required at the expansion stage of the business because that time helps us to build connections and can lead to more customer base ….’.*

Reference 4- *‘… While networking is mainly in the initial stage it is required to be on the network, you need networking at every stage because networking is important for all stages to stay connected with others. For example, Flipkart's business started with networking, and it continues with networking for its sales ….’.*

### 4.4 Theme 4: Role of the past work experience of an entrepreneur

**Figure 4.**
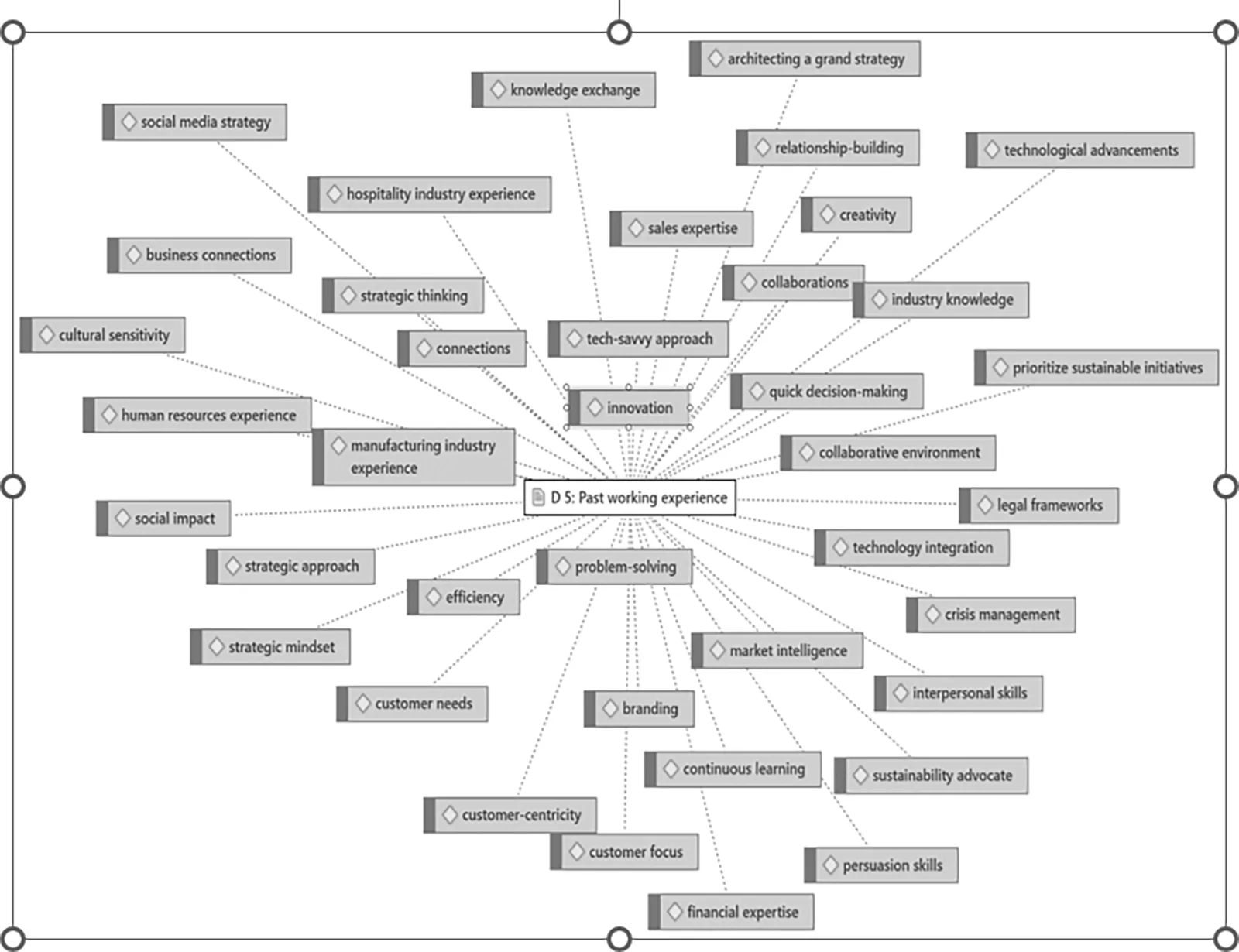
Role of past work experience in an entrepreneur’s skills and knowledge. Source: ATLAS. ti thematic map.

Based on the information gathered from the interviews, the thematic map presented in Figure 4 is included in the list of sources that follow. Past working experiences of entrepreneurs shape their interactions with people. These perspectives encompass a wide array of skills and attributes, each contributing uniquely to their ability to connect and thrive in their ventures. These entrepreneurs excel in building meaningful relationships with stakeholders and fostering trust and collaboration, and their strong networking abilities facilitate access to resources and opportunities. Sales and customer support skills ensure exceptional service delivery and customer satisfaction, while crisis management abilities mitigate risks and preserve business continuity. Agility enables them to adapt swiftly to changing market dynamics, while sustainability initiatives promote responsible business practices and environmental stewardship.

Reference 1- *‘… Before becoming an entrepreneur, I was working in the banking sector due to enormous pressure and overworking I quit the job, and during that time I learned many things like—a strategist approach to tackling any situation be it interacting with investors or developing the market strategy ….’.*

Reference 2- *‘My salesperson job helped me to learn about customer interaction, and I applied this to my startup to make it a more customer-driven approach ….’.*

Reference 3- *‘….My learnings from the previous job helps me with the right decision-making and problem-solving skills ….’.*

Reference 4- *‘….I was a travel blogger before starting my startup, so I have no experience working, but I got to know more about dealing with people, so that experience gives me some kind of comfort in my startup ….’.*

## 5. Discussion

This qualitative analysis revealed that networking is an important tool that ranges from accessing new opportunities and resources to fostering adaptability, innovation, and brand building, which aligns with previous studies ( Ford & Mouzas, 2013; Ferreira *et al*., 2022). This study further supports the importance of networking as a crucial tool for accessing new opportunities and resources and fostering adaptability, innovation, and brand reinforcement. Additionally, the study highlights the alignment of these findings with previous studies that emphasize the significance of networking in achieving business success ( Ford & Mouzas, 2013). Networking is a multifaceted tool that not only facilitates career growth and collaborative problem-solving but also plays a vital role in community building, competitor analysis, and continuous learning. Crucially, during times of crisis, the strength of one's network becomes most evident as members rally to offer expert counsel, unwavering moral support, and crucial connections to specialist resources. This collaborative effort helps in a problem-solving approach that opens doors to unforeseen opportunities and bolsters the resilience of startups. It also drives the benefits of networking, such as accessing specialized expertise, maintaining connections, and facilitating ongoing personal and professional development ( Ferreira *et al*., 2022). By understanding the significance of each aspect, individuals and businesses can prioritize their networking efforts effectively to achieve specific goals, whether by expanding their brand's visibility, securing funding, or fostering collaborative partnerships.

Formal networking can provide startups with credibility and visibility within their industry, enhancing their reputation and attracting potential customers or clients ( Klerk & Saayman, 2012; Sharafizad, 2014; Stuart & Sorenson, 2007). This research revealed that formal networking serves as the lifeblood of startup success, acting as a multifaceted catalyst for growth and resilience. It operates as a dynamic engine that propels ventures forward, facilitating pivotal roles such as forging strategic partnerships, securing crucial investments, expanding customer bases, and nurturing a culture of innovation. By engaging with fellow entrepreneurs and ex-bosses, individuals gain not only invaluable support but also access to a wealth of insights and potential avenues for investment. Additionally, some participants have argued that business coaches also emerge as significant people in networking circles, offering valuable mentorship and guidance to entrepreneurs, which aligns with the past literature ( Ferreira *et al*., 2022). Guidance for personal and professional development emanates from a plethora of sources, including seasoned industry veterans, dedicated mentors, and vibrant online communities. This study underscores the importance of strategic networking efforts, which guide individuals and businesses toward avenues that offer the greatest potential impact and opportunities for growth.

It has also been found that networking is moderately important at the initial/early stage, serving as a critical means to establish initial connections and garner support from the entrepreneurial community. Conversely, some participants feel that as businesses progress into the expansion and growth stages, networking becomes increasingly important, with higher densities indicating widespread adoption to access new markets, forming alliances, and sustaining scalability, which aligns with the findings of some researchers ( Ford & Mouzas, 2013; Pittaway *et al*., 2004). While the importance of networking persists throughout a venture's lifecycle, its significance is particularly pronounced during nascent stages. Here, it serves as a vital conduit for market feedback, the formation of strategic alliances, and the navigation of early-stage challenges, laying the groundwork for sustained growth and success. Notably, networking during the decline stage, while relatively important, exhibits a lower prevalence, possibly indicating a shift in focus toward internal restructuring and crisis management. The acknowledgment of the stages of networking itself underscores the awareness of its dynamic nature throughout the business lifecycle.

Drawing upon past experiences, entrepreneurs use many different skills they have learned from past jobs to run their businesses. They learnt to communicate well, lead confidently, and understand the market they were in. They learnt these skills by talking to others at events, researching the market, and receiving advice from mentors. This helps them make smart choices for their business. These findings align with previous research showing that prior work experience plays a significant role in an entrepreneur's skills and knowledge, influencing their ability to recognize and act on opportunities, manage new ventures, and address the changing role of a business founder ( Martin and Smith, 2010). This experience allows them to identify opportunities, anticipate challenges, and make informed decisions ( Dorcas *et al*., 2021).

## 6. Managerial Implications

Entrepreneurs are dependent mainly on their social networks to obtain crucial resources, information, and opportunities, underscoring the pivotal role that social connections play in entrepreneurship. Networking helps startups expand their reach, access valuable resources, build partnerships, gain insights, and stay abreast of industry trends. Since networking is essential at all stages of a startup, the nature and focus of the networks may change as the startup progresses, and small and new firms should focus on building networks along with their preparation of business plans. These early network relationships are crucial for sustained innovation in small entrepreneurial firms.

Another aspect of startup success is past work experience, which shapes an entrepreneur's skills and knowledge. Entrepreneurs who have previously worked in similar industries or roles gain valuable insights, expertise, and a deep understanding of market dynamics. If an entrepreneur has previous work experience, he or she can use these contacts and connections for partnerships, funding, and mentorship. If he/she is a novice, the experience of his/her team or social/informal networks can serve as a valuable resource for knowledge sharing, collaboration, and accessing necessary resources. The strategic use of these connections helps in building credibility and gaining the trust of potential investors, clients, and partners. Since entrepreneurs with varied work experience exhibit greater entrepreneurial skills relevant to starting and growing a firm, starting a venture in a known industry increases the likelihood of success. The supportive environment of network members can be useful for improving interpersonal skills, communication, problem-solving, customer-centricity, continuous learning, strategic thinking, cultural sensitivity, global connections, collaboration, crisis management, and so on.

## 7. Research Limitations and Future Scope

The empirical analysis in this study has limitations due to the relatively small sample size, so there might be more to learn by studying more people and using different research methods. To address this constraint, future research should adopt a mixed-methodological approach with a more extensive sample to enhance generalizability. Future research could explore additional factors, including family background, skills, recognition, and self-realization, to enrich the understanding of their impact on their startups. There is scope for utilizing larger datasets encompassing a wide range of startups to investigate cross-country variation in networking behavior. There is a need to look at how networking differs among men and women entrepreneurs. Additionally, there is a lack of research on the cultural and gender-related challenges that entrepreneurs and startups may face in networking. Exploring these challenges can provide valuable insights into the barriers that diverse entrepreneurs and startups encounter in networking and inform the development of inclusive strategies to overcome them.

## 8. Conclusion

The qualitative analysis of networking experiences was conducted among entrepreneurs through semi-structured interviews in which four main themes were explored: the role of networking in startup success, supportive network members, crucial stages of networking, and the influence of past work experience on entrepreneurial skills. This study reaffirms the significance of networking in achieving business success. Formal networking has emerged as a critical aspect of startup success, providing credibility, visibility, and opportunities for growth. While networking remains important throughout the business lifecycle, its significance is particularly found in the early stages because it lays the foundation for sustained growth by facilitating market feedback, strategic alliances, and early-stage challenges navigation. Past work experience plays a significant role in shaping entrepreneurial skills and knowledge, influencing the ability to recognize opportunities, manage ventures, and make informed decisions. By understanding and leveraging the dynamics of networking, entrepreneurs can effectively navigate challenges, capitalize on opportunities, and build successful startups.

## Ethical approval and consent

Ethical approval was provided by the Institutional Ethical Committee of Manipal (MAHE) for this research endeavor, which was carried out as part of a Ph.D. study, and approval was given on 14 January 2023, under reference number IEC-2022-328. It ensures that all study participants provided informed consent in strict accordance with ethical norms. Before providing consent, participants were required to complete a consent form that was intended to ensure that they were fully informed about the study's objectives, methods, and any risks and advantages of participating.

Participants have provided written consent in the form of yes and no statements in the data sheet while collecting information for interviews (transcription applies only to interviews). They will also consent to the anonymous use of data for publication purposes.

All confidential data has been secured by password-protected computers which are accessible only to the research team members of this project. Personal data will undergo anonymisation, with identifying details replaced by unique alphanumeric codes. During the transcription process, audio recordings are anonymized, which ensures that all personally identifiable information is removed before utilizing analytical software.

## Author contributions

The contributions of each author to this study are as follows:

Sheetal Singh - Substantial contributions to the conception or design of the work, searching articles, conceptualization, analysis of data, first draft of the paper write-up, and interpretation of data for the work.

Dr. Basri Savitha - Substantial contributions to the conceptualization, analysis of the data, and review of the manuscript and final draft of the paper.

## Data Availability

The raw data for this study are not available due to the privacy concerns of the participants. Only the rough version of the interview transcript has been provided as supplementary material. If required, readers can email the first author for further information on the study. This project contains the following extended data: Figshare: Dataset for An analysis on understanding entrepreneurial networking behavior among Indian startups, https://doi.org/10.6084/m9.figshare.27968547.v2 ( Singh, S., 2024).
• A questionnaire and demographic profile on understanding entrepreneurial networking behavior among Indian startups (quantitative questionnaire) • Transcript as a supplementary material (rough data) Data are available under the terms of the Creative Commons Attribution 4.0 International license (CC-BY 4.0).
