# Supplementary material for: Understanding networking behaviors among Indian startups: A qualitative study
Source: F1000Res. 2026 Jul 11;14:278. Originally published 2025 Mar 10. [Version 3] doi: 10.12688/f1000research.159752.3 (PMC13554736; doi:10.12688/f1000research.159752.3)
Supplement: Peer review history

# Reviewer report: Reviewer response for version 3

**DOI:** 10.5256/f1000research.205055.r500550

**Published:** 2026-07-21

**Authors:** Suja Pradeep

**Affiliations:** 1 College of Business, Dept of Accounting and Finance, Liwa College, Abu Dhabi, United Arab Emirates

Having reviewed the authors’ responses and the updated manuscript, I am pleased to see that they have comprehensively addressed the main methodological and theoretical concerns raised in the previous review round. The amendments are fully sufficient and I am happy to update my recommendation status to "Approved."

Is the work clearly and accurately presented and does it cite the current literature?

Yes

If applicable, is the statistical analysis and its interpretation appropriate?

Yes

Are all the source data underlying the results available to ensure full reproducibility?

No

Is the study design appropriate and is the work technically sound?

Yes

Are the conclusions drawn adequately supported by the results?

Yes

Are sufficient details of methods and analysis provided to allow replication by others?

Yes

Reviewer Expertise:

Digital Transformation, Entrepreneurship, Sustainable Consumption Behavior, Circular Economy.

I confirm that I have read this submission and believe that I have an appropriate level of expertise to confirm that it is of an acceptable scientific standard.

# Reviewer report: Reviewer response for version 2

**DOI:** 10.5256/f1000research.189893.r475296

**Published:** 2026-05-13

**Authors:** Suja Pradeep

**Affiliations:** 1 College of Business, Dept of Accounting and Finance, Liwa College, Abu Dhabi, United Arab Emirates

This study examines the networking experiences and behaviors of 36 startup entrepreneurs from the startup ecosystem of Bangalore and Delhi regions of India. Using Social Network Theory, the study elaborates on four key themes: networking and its impact on startup success, networking championing, critical periods for networking, and the impact of previous work experience of the entrepreneur. Results show that there is a strong professional networking advantage from former working contacts, whereas the informal familial and friendly framework is a strong working support during crises. Further, the study also indicates a growing dependency for the resources mobilization on the digital platforms after the COVID-19 pandemic. An issue of Overgeneralization is noted: The study provides important insights into the networking practices of startups, but there is a wide gap between broad title (i.e. Indian Startups) and the narrow scope of the data. The sample is concentrated on Bangalore and Delhi and restricted to fintech and healthtech sectors. Hence, the results may not be representative of the wider Indian ecosystem. I suggest either the title be narrowed to indicate this specific geographic and sectoral focus, or a strong discussion section acknowledging the exclusion of Tier-2 and Tier-3 cities as a major limitation to the generalisability of the study. Also suggesting to integrate Social Network Theory with India-specific factors (like caste or family-led business structures) in the *Results* section, rather than just in the *Literature Review*.

Is the work clearly and accurately presented and does it cite the current literature?

Yes

If applicable, is the statistical analysis and its interpretation appropriate?

Yes

Are all the source data underlying the results available to ensure full reproducibility?

No

Is the study design appropriate and is the work technically sound?

Yes

Are the conclusions drawn adequately supported by the results?

Yes

Are sufficient details of methods and analysis provided to allow replication by others?

Yes

Reviewer Expertise:

Digital Transformation, Entrepreneurship, Sustainable Consumption Behavior, Circular Economy.

I confirm that I have read this submission and believe that I have an appropriate level of expertise to confirm that it is of an acceptable scientific standard, however I have significant reservations, as outlined above.

# Reviewer report: Reviewer response for version 2

**DOI:** 10.5256/f1000research.189893.r432676

**Published:** 2026-01-29

**Authors:** Johannes Baptista Halik, Petrus Peleng Roreng

**Affiliations:** 1 Management, Paulus Christian University of Indonesia, Makassar, South Sulawesi, Indonesia; 2 Accounting, Paulus Indonesian Christian University (Ringgold ID: 185805), Makassar, South Sulawesi, Indonesia

The manuscript offers a rich qualitative account of networking behaviors among Indian startups and demonstrates commendable methodological transparency, particularly in its use of semi-structured interviews, thematic analysis, and ethical disclosure. The contextual grounding in post-COVID India, with attention to FinTech and HealthTech sectors, further strengthens its empirical relevance. However, despite these merits, several **major conceptual and analytical limitations** currently constrain the manuscript’s contribution to the international entrepreneurship and social network literature.

 First, **the theoretical contribution remains insufficiently articulated and largely confirmatory rather than generative**. Although the study is explicitly framed within Social Network Theory, the empirical findings predominantly reiterate well-established concepts such as the importance of strong and weak ties, early-stage reliance on personal networks, and the role of prior work experience in network activation. These insights, while empirically sound, do not substantially extend, refine, or challenge existing theoretical frameworks. The manuscript would benefit from a clearer articulation of how the Indian sociocultural context—particularly caste dynamics, family embeddedness, and post-pandemic digital networking—either modifies or problematizes classical network assumptions. At present, the reader is left with the impression that context is added descriptively rather than leveraged analytically to advance theory.

 Second, **the analysis remains predominantly descriptive, with limited depth in analytical abstraction**. Participant quotations are used effectively to illustrate themes; however, they rarely function as vehicles for theory-building or conceptual tension. Although the authors state that contradictions and negative cases were examined, these are not sufficiently integrated into the analytical core of the paper. As a result, networking is still portrayed primarily as a beneficial mechanism, while its potential constraining, exclusionary, or path-dependent effects remain underdeveloped. A more critical engagement with instances where networking failed, produced lock-in effects, or restricted innovation would significantly strengthen the analytical rigor of the study.

 Third, **the manuscript’s claims exceed the empirical scope of the data**. While the study repeatedly refers to “Indian startups,” the sample is limited to 36 entrepreneurs operating in Bangalore and Delhi, two highly privileged and resource-rich ecosystems. This raises concerns regarding analytical overgeneralization, particularly given the known heterogeneity of India’s startup landscape across regions, sectors, and institutional environments. The absence of entrepreneurs from tier-2 and tier-3 cities, informal sectors, or non-tech industries limits the extent to which the findings can be interpreted as representative of Indian startups more broadly. A more cautious positioning of the study’s scope is therefore warranted.

 Fourth, **the gender analysis, although valuable, remains under-theorized and analytically shallow**. While gender-disaggregated findings are reported, the discussion largely frames women entrepreneurs in terms of constraints and barriers, with limited attention to agency, adaptive strategies, or alternative forms of network mobilization. Moreover, the analysis does not sufficiently engage with intersectional dynamics, such as how gender interacts with caste, class, or regional location in shaping access to networks. This limits the study’s potential contribution to the growing literature on gendered social capital in entrepreneurship.

 Finally, **the literature review, despite being extensive and up-to-date, is not sufficiently integrated into the empirical analysis**. A large number of recent references are cited, but many are not explicitly mobilized to interpret findings, generate propositions, or position the study within ongoing scholarly debates. As a result, the review functions more as a comprehensive background section than as a theoretical scaffold guiding analysis. Greater selectivity and tighter theory–data dialogue would enhance conceptual clarity and coherence.

 In its current form, the manuscript represents a solid qualitative exploration of entrepreneurial networking within a specific Indian context. However, without a stronger theoretical positioning, deeper analytical engagement with contradictions and failures, and a more cautious alignment between data and claims, its contribution remains primarily contextual rather than conceptual. Addressing these issues would significantly improve the manuscript’s suitability for a high-impact, Scopus-indexed international journal.

Is the work clearly and accurately presented and does it cite the current literature?

Partly

If applicable, is the statistical analysis and its interpretation appropriate?

Not applicable

Are all the source data underlying the results available to ensure full reproducibility?

Partly

Is the study design appropriate and is the work technically sound?

Partly

Are the conclusions drawn adequately supported by the results?

Partly

Are sufficient details of methods and analysis provided to allow replication by others?

Partly

Reviewer Expertise:

Entrepreneurship, Digital Marketing, Small and Medium Enterprises (SMEs), Innovation Management, and Qualitative/Quantitative Research Methods (SEM-PLS).

We confirm that we have read this submission and believe that we have an appropriate level of expertise to confirm that it is of an acceptable scientific standard, however we have significant reservations, as outlined above.

## References

[1] : Strategic Distribution, Digital Marketing, and Open Innovation: Toward Integrated Capability Development for SME Performance. *Asia Marketing Journal* .2026;27(4) : 10.53728/2765-6500.1665 355-371 10.53728/2765-6500.1665

# Reviewer report: Reviewer response for version 1

**DOI:** 10.5256/f1000research.175523.r406623

**Published:** 2025-10-29

**Authors:** Muhammad Hasan

**Affiliations:** 1 Universitas Negeri Makassar, Makassar, Indonesia

This paper, *“Understanding Networking Behaviors among Indian Startups: A Qualitative Study,”* addresses a timely and relevant topic, given India’s vibrant startup ecosystem and the growing scholarly interest in entrepreneurial networking. The qualitative approach using semi-structured interviews provides valuable insights into the lived experiences of entrepreneurs and highlights the role of networking across stages of startup growth. However, while the manuscript demonstrates clear organization and ethical rigor, it would benefit from stronger theoretical framing, methodological transparency, and contextual depth.

 The **introduction and literature review** provide a general overview of networking and its importance but rely heavily on dated references (e.g., Witt, 2004; Stuart & Sorenson, 2007) and lack discussion of recent developments such as post-COVID digital networking, virtual ecosystems, or gendered networking patterns. The review would be strengthened by incorporating contemporary sources (2023–2025) and clarifying how India’s cultural, institutional, and regional factors—such as caste, family-led business traditions, and digital infrastructure—uniquely shape entrepreneurial networking. Doing so would enhance the paper’s originality and contribution.

 The **methodology** section is sound in its use of semi-structured interviews and thematic analysis via ATLAS.ti, but important details are missing. The paper should explain how participants were recruited, how saturation was reached, and whether the interview guide was validated or piloted. The coding process should be elaborated—detailing codebook development, intercoder reliability, and theme generation. Including reflexivity (the researchers’ positionality and how their interpretations may influence analysis) would enhance credibility and trustworthiness. Providing sample coded excerpts and a thematic map appendix would also help demonstrate analytical rigor.

 The **findings and discussion** clearly identify four themes, each supported by participant quotes. However, the interpretation remains largely descriptive. The paper should delve deeper into analytical contrasts—for example, exploring when networking *fails* or when over-networking creates dependency or fatigue. Integrating formal network theory (e.g., Granovetter’s “strength of weak ties,” Burt’s “structural holes,” or Nahapiet & Ghoshal’s “social capital”) could provide conceptual coherence and connect the themes to broader entrepreneurship theory. Additionally, distinguishing more sharply between early-stage and growth-stage networking behaviors would clarify nuances within the data.

 The **conclusion and managerial implications** appropriately summarize the main points and emphasize the role of prior experience, but they tend to overgeneralize. Claims such as “networking is important at all stages” should be nuanced to reflect participant diversity. It would also be valuable to expand the policy and ecosystem implications—for instance, how incubators, government initiatives, or university entrepreneurship cells can foster inclusive networking, especially for women or rural entrepreneurs. Finally, data transparency should be improved: while privacy must be respected, anonymized excerpts or controlled-access transcripts could enhance reproducibility and scholarly trust.

 In summary, this study presents a relevant and potentially impactful contribution to understanding entrepreneurial networking in emerging economies. By updating the literature, deepening theoretical grounding, clarifying methodological rigor, and contextualizing India’s unique ecosystem, the authors can substantially enhance the paper’s scholarly value and international appeal.

Is the work clearly and accurately presented and does it cite the current literature?

Partly

If applicable, is the statistical analysis and its interpretation appropriate?

Partly

Are all the source data underlying the results available to ensure full reproducibility?

Partly

Is the study design appropriate and is the work technically sound?

Partly

Are the conclusions drawn adequately supported by the results?

Partly

Are sufficient details of methods and analysis provided to allow replication by others?

Partly

Reviewer Expertise:

Entrepreneurship EducationEconomics EducationEconomic LiteracyKnowledge Transfer

I confirm that I have read this submission and believe that I have an appropriate level of expertise to confirm that it is of an acceptable scientific standard, however I have significant reservations, as outlined above.

# Reviewer report: Reviewer response for version 1

**DOI:** 10.5256/f1000research.175523.r406619

**Published:** 2025-10-02

**Authors:** Johannes Baptista Halik

**Affiliations:** 1 Management, Paulus Christian University of Indonesia, Makassar, South Sulawesi, Indonesia

Peer Review Report

**Article title:** *Understanding networking behaviors among Indian startups: A qualitative study*

**Authors:** Singh S. & Basri S.

**Journal:** F1000Research, 2025

**Summary of the Article**

 This study investigates the networking behaviors of Indian startup entrepreneurs through a qualitative design. Semi-structured interviews were conducted with 36 entrepreneurs based primarily in Bangalore and Delhi. Thematic analysis of the interview data, supported by ATLAS.ti, revealed four overarching themes:

1. The role of networking in startup success.
2. Supportive environments and people in networking.
3. Crucial stages of networking across the business lifecycle.
4. The influence of prior work experience on networking behaviors.

The authors argue that networking is pivotal for startup success, particularly in early stages, and that prior experience enhances entrepreneurs’ ability to build and leverage networks. The paper concludes with managerial implications, emphasizing the importance of formal/informal networks, and identifies avenues for future research.

 Evaluation Against Review Criteria

**Is the work clearly and accurately presented and does it cite the current literature?**

**Answer: Partly**

- **Strengths:** The manuscript is generally well-written, with a logical structure and clear expression. The literature review covers foundational ideas such as formal/informal networking and strong/weak ties.
- **Weaknesses:**
  - Over-reliance on dated sources (e.g., Witt, 2004; Stuart & Sorenson, 2007). Only 8 of 38 references are post-2020, which undermines the claim of addressing a “gap.”
  - Contextualization is superficial. While India is an important entrepreneurial ecosystem, the paper does not adequately explain what is unique about Indian startups (e.g., caste, regional ecosystems, family-led businesses).
  - Findings such as “networking aids crisis support” echo prior work (e.g., Albourini et al., 2020) without offering strong novelty.

**Suggestions:** Update the literature review with 2023–2025 studies, especially post-COVID networking research. Contextualize the study in India more explicitly (e.g., cultural, gender, and regional factors). Highlight contributions beyond confirming established findings.

**Is the study design appropriate and is the work technically sound?**

**Suggestions:**

- Justify why Bangalore/Delhi and Fintech/Healthtech were prioritized, or acknowledge this as a limitation.
- Incorporate network theory explicitly to strengthen the conceptual foundation.
- Provide details on the coding process: codebook development, coder agreement, and how disagreements were resolved.
- Include a reflexivity statement to address researcher positionality.

**Are sufficient details of methods and analysis provided to allow replication by others?**

**Suggestions:**

- Expand the methods section with details on sampling, recruitment, and demographics.
- Provide an appendix with sample codes and excerpts showing the transition from codes to themes.
- Explain how data saturation was determined.

**If applicable, is the statistical analysis and its interpretation appropriate?**

- The study is purely qualitative and does not involve statistical analysis. The use of thematic analysis is appropriate for the design.

**Are all the source data underlying the results available to ensure full reproducibility?**

**Suggestions:** Consider depositing anonymized transcripts or excerpts (e.g., with identifiers removed) in a secure repository with controlled access. This would balance ethical concerns with transparency.

**Are the conclusions drawn adequately supported by the results?**

**Suggestions:**

- Revise conclusions to reflect nuances and contradictions in the data.
- Avoid overgeneralizations—differentiate clearly between early-stage and later-stage networking dynamics.
- Explicitly acknowledge limitations in novelty and highlight where new insights (e.g., Indian-specific dynamics) emerge.

**Overall Recommendation**

 The study addresses an important topic in an underexplored context and offers practical insights for startup entrepreneurs. However, significant revisions are needed to strengthen its academic rigor and contribution. With improvements in theoretical framing, methodological transparency, and contextual justification, the article has the potential to make a valuable contribution to the entrepreneurship and networking literature.

**Recommendation: Major Revisions**

Is the work clearly and accurately presented and does it cite the current literature?

Partly

If applicable, is the statistical analysis and its interpretation appropriate?

Not applicable

Are all the source data underlying the results available to ensure full reproducibility?

Partly

Is the study design appropriate and is the work technically sound?

Partly

Are the conclusions drawn adequately supported by the results?

Partly

Are sufficient details of methods and analysis provided to allow replication by others?

Partly

Reviewer Expertise:

Entrepreneurship, Digital Marketing, Small and Medium Enterprises (SMEs), Innovation Management, and Qualitative/Quantitative Research Methods (SEM-PLS).

I confirm that I have read this submission and believe that I have an appropriate level of expertise to confirm that it is of an acceptable scientific standard, however I have significant reservations, as outlined above.

# Reviewer report: Reviewer response for version 1

**DOI:** 10.5256/f1000research.175523.r394399

**Published:** 2025-08-14

**Authors:** Shah Muhammad Kamran

**Affiliations:** 1 Mehran University of Engineering and Technology, Sindh, Pakistan

"Understanding networking behaviors among Indian startups: A qualitative study"

 Dear Editor,

 Thank you for the opportunity to review this manuscript. The topic is highly relevant, especially given India’s growing startup ecosystem, and addresses a gap in qualitative research on entrepreneurial networking beyond early-stage ventures.

 This research study examines the networking behavior among Indian entrepreneurial startups (36) located in Bangalore and Delhi. Applying thematic analysis through semi-structured interviews, four themes were identified (1) networking’s role in startup success, (2) supportive environments/people, (3) crucial stages for networking, and (4) the influence of past work experience. Study emphasize on the importance of networking, for crisis support, opportunities, and resources sharing/pooling, especially at early stages. It further highlights the importance of formal and informal ties and kind of prior experience.

 Final Remarks

 Though the study addresses a current topic but the identified themes are so general and obvious, furthermore, significant methodological and contextual limitations reduce its academic impact. Major Revisions are required to justify its novelty, enhance rigor, and ensure ethical reproducibility. With significant change this manuscript can be improved and contribute in the existing body of literature in the field of entrepreneurial research.

**Recommendation**: **Major Revisions**

 Dear Author(s),

 This study about networking behavior among early-stage startups in India is addressing a timely topic. Though the manuscript is well written and articulated. However, there is always room for improvement. The following are comments and suggestions for the betterment of the manuscript;

 Strengths

1. **Relevant Topic**: Focuses on understudied networking dynamics in a key emerging economy, like India.
2. **Clear Structure**: Well-organized sections (intro, methods, results, discussion).
3. **Thematic Richness**: Identifies nuanced themes (e.g., crisis support, stage-specific networking).
4. **Practical Implications**: Offers actionable insights for entrepreneurs (e.g., leveraging past experience, building formal/informal networks).
5. **Ethical Rigor**: Institutional approval and participant consent documented.

**Critical Assessment & Weaknesses**

 1. **Clarity, Accuracy & Literature Integration**

- **Strengths**: Writing is generally clear; literature review covers foundational concepts (e.g., strong/weak ties, formal/informal networks).
- **Weaknesses**:
  -
    - **Superficial Contextualization**: Fails to justify *why India* warrants specific study. Cultural nuances (e.g., family-led businesses) are ignored, weakening the contribution.
    - **Dated Citations**: Over-reliance on pre-2020 sources (e.g., Witt 2004, Stuart & Sorenson 2007) despite claiming a "gap" in recent literature. Only 8/38 references are post-2020.
    - **Redundant Claims**: Findings (e.g., "networking aids crisis support") echo prior work (e.g., Albourini et al. 2020) without novel insights.

2. **Study Design & Academic Merit**

- **Strengths**: Qualitative design is appropriate for exploring subjective experiences.
- **Weaknesses**:
  -
    - **Sampling Flaws**:
    - **Demographic Gap**: 75% male participants; gender dynamics in networking remain unexplored.
    - **Regional Bias: **Though the title states India, only two regions, Bangalore/Delhi entrepreneurs (∼60% of Indian startups) are studied. Justification is needed for the exclusion of other Rural/underrepresented regions.
    - **Sector Imbalance**: Heavy focus on Fintech/Healthtech (no justification). Ignores manufacturing/social enterprises.
    - **Lack of Theoretical Framework**: No grounding in network theory (e.g., Burt’s structural holes, Granovetter’s strength of weak ties).

3. **Methodological Transparency & Replicability**

- **Critical Weaknesses**:
  -
    - **Interview Guide**: Provided (Table 1) but lacks validation (e.g., pilot testing, expert review).
    - **Coding Process**: Vague description. How were themes derived? No inter-coder reliability measures, risking bias.
    - **Analytical Rigor**: Claims "thematic analysis" but omits critical steps (e.g., reflexivity journal, divergent case analysis).
    - **Software Use**: Mentions ATLAS.ti but no details on coding structure/queries.

4. **Statistical Analysis**

- Not applicable (qualitative study).

5. **Data Availability & Reproducibility**

- **Unacceptable Limitations**:
- Raw data "not available due to privacy concerns" (Section 8). Only "rough transcripts" shared.
- Anonymized data could be archived (e.g., via controlled access). Current stance undermines reproducibility and ethical transparency.

6. **Conclusions Supported by Results?**

- **Partial Support**: Conclusions align with themes but overstate novelty:
  - Theme 4 ("past experience") is well-supported by quotes (e.g., banking skills aiding strategy). *Note: “Past Experience” can be replaced with “Prior Experience” as experience always comes with the past.*
  - **Overgeneralization**: Claims about "all stages" of networking (Ref. 4, p.9) contradict participant data (e.g., Ref. 2, p.8: reduced networking post-establishment).
  - **Ignored Contradictions**: No discussion of cases where networking *failed* (e.g., incubators’ poor external support, p.4).

**Suggestions for Improvement**

1. **Theory & Context**:
  - Ground in network theory (e.g., social capital framework).
  - Integrate India-specific socio-economic factors (e.g., caste, regional ecosystems).
2. **Methods**:
  - Justify sampling strategy (e.g., purposive sampling for diversity).
  - Include gender-disaggregated analysis.
  - Detail coding process: codebook development, resolution of disagreements.
3. **Analysis**:
  - Use quotes more critically (e.g., highlight contradictions).
  - Explore negative cases (e.g., networking that *hindered* growth).
4. **Data Sharing**:
  - Archive anonymized transcripts/audio via secure repositories (e.g., UK Data Service) with access protocols.
5. **Literature**:
  - Update references (e.g., cite 2023–2025 studies on post-COVID networking).
  - Compare findings with similar economies (e.g., Brazil, Nigeria, South Africa).
6. **Implications**:
  - Add policy recommendations (e.g., government-led networking hubs for women/rural entrepreneurs).

Is the work clearly and accurately presented and does it cite the current literature?

Partly

If applicable, is the statistical analysis and its interpretation appropriate?

Not applicable

Are all the source data underlying the results available to ensure full reproducibility?

Partly

Is the study design appropriate and is the work technically sound?

Partly

Are the conclusions drawn adequately supported by the results?

Partly

Are sufficient details of methods and analysis provided to allow replication by others?

No

Reviewer Expertise:

Economics, Qualitative and Quantitative Research, Entrepreneurship, Analytics

I confirm that I have read this submission and believe that I have an appropriate level of expertise to confirm that it is of an acceptable scientific standard, however I have significant reservations, as outlined above.
